# Mediators of quality of life change in people with severe psychotic disorders treated in integrated care: ACCESS II study

**DOI:** 10.1192/j.eurpsy.2022.2332

**Published:** 2022-11-04

**Authors:** Romy Schröter, Martin Lambert, Anja Rohenkohl, Vivien Kraft, Friederike Rühl, Daniel Luedecke, Jürgen Gallinat, Anne Karow, Stefanie J. Schmidt

**Affiliations:** 1 Centre for Psychosis and Bipolar Disorders, Department of Psychiatry and Psychotherapy, Centre for Psychosocial Medicine, University Medical Center Hamburg-Eppendorf, Hamburg, Germany; 2 Department of Clinical Psychology and Psychotherapy, University of Bern, 3012 Bern, Switzerland

**Keywords:** Assertive Community Treatment, bipolar disorder, quality of life, schizophrenia, severe mental illness

## Abstract

**Background:**

Patients with severe psychotic disorders exhibit a severely reduced quality of life (QoL) at all stages of the disease. Integrated care often led to an improvement in QoL. However, the specific mediators of QoL change are not yet well understood.

**Methods:**

The ACCESS II study is a prospective, long-term study investigating the effectiveness of an integrated care program for people with severe psychotic disorders (IC-TACT) that includes Therapeutic Assertive Community Treatment within a care network of in- and outpatient services at the University Medical Center Hamburg-Eppendorf, Germany. We examined longitudinal associations between QoL and the hypothesized mediators of change (i.e., negative symptoms, depression, and anxiety), using cross-lagged panel models.

**Results:**

The sample includes 418 severely ill patients treated in IC-TACT for at least 1 year. QoL increased, whereas symptom severity decreased significantly from baseline to 6-month follow-up (*p*-values ≤ 0.001), and remained stable until 12-month follow-up. QoL and symptom severity demonstrated significant auto-correlated effects and significant cross-lagged effects from QoL at baseline to negative symptoms (6 months, *β* = −0.20, *p* < 0.001) to QoL (12 months, *β* = −0.19, *p* < 0.01) resulting in a significant indirect, mediated effect. Additionally, negative symptoms after 6 months had a significant effect on the severity of depression after 12 months (*β* = 0.13, *p* < 0.05).

**Conclusions:**

Negative symptoms appear to represent an important mechanism of change in IC-TACT indicating that improvement of QoL could potentially be achieved through optimized intervention on negative symptoms. Moreover, this may lead to a reduction in the severity of depression after 12 months.

## Introduction

Quality of life (QoL) has become an important issue in the care of people with mental illness. Major reasons include the increasing community-based and patient-centered care, the importance of subjective well-being, and the acceptance of QoL as an important criterion for treatment success [1]. Although there is no universal definition of QoL, it is generally accepted that it contains both objective (e.g., mental and physical health) and subjective (e.g., feeling of well-being and satisfaction) dimensions [2, 3].

Patients with psychotic disorders, especially those diagnosed with schizophrenia or those who meet the criteria for severe mental illness (SMI), exhibit a severely reduced QoL at all stages of the disease. Systematic reviews and meta-analyses have shown that patients at risk for the development of psychosis [4] and during the early [5] and long-term phases [6] have a reduced QoL. The main mediating factors comprise poor mental and physical health, depression, anxiety, severity of illness, coping, problems in social relationships, and environmental domains such as living circumstances or finances [6].

Evidence-based care including evident care models (Early Intervention Services [7–9] and Assertive Community Treatment (ACT) [10]) including evident treatment components (e.g., pharmacotherapy, cognitive-behavioral therapy, and social and somatic interventions [7, 9, 11]) often led to an improvement in QoL. However, with regard to mental health as one of the key factors affecting QoL, the specific mechanism of change that make ACT effective with regard to QoL are not yet well understood [12].

The identification of such mediators (mechanisms) of change requires the study of intervening variables that account for the effect of a specific treatment, such as IC-ACT, on the outcome of interest [12]. Possible mediators linking the treatment content to the improvement of QoL are levels of anxiety, depression, and negative symptoms as these have been demonstrated to respond to IC-ACT [8, 13, 14] and to be associated with QoL [6], both cross-sectionally and longitudinally [6].

In line with these results, a recent study demonstrated that treatment-induced effects of IC-TACT on QoL after 12 months were mediated by changes in anxiety, depressive, and negative symptoms [12]. More precisely, changes in QoL were achieved by two pathways: one pathway leading from changes in negative symptoms to depressive symptoms and a second one through changes in anxiety. However, in the cited study change scores of all mediators and QoL between baseline and follow-up assessment were used. This does not allow any conclusion about the temporal order between these variables which is inherently postulated in a mediation model, that is, anxiety, depressive, and negative symptoms are predictive of QoL and not vice versa. Thus, it is required to investigate both mediators and outcome variable (QoL) at repeated measures over time to disentangle cause and effect by taking reciprocal effects into account. Additionally, such a procedure would provide a more fine-grained understanding of potential mechanisms of change of ACT as it also allows to disentangle the effects of mediators by investigating at which time-point a mediator exerts its largest effect on other mediators as well as on the outcome of interest [15].

Another limitation refers to the fact that most studies investigating the mechanisms of change of ACT so far used standard regression procedures not taking the stability of symptom levels and QoL over time into account. This may have led to an overestimation of the longitudinal association between two variables due to the high stability of these constructs in terms of high auto-correlations across time. Furthermore, these results may have been biased by not taking cross-sectional associations between symptom levels and QoL measured at the same time point into account.

This may have led to an overestimation of the longitudinal association between two variables due to the high stability of these constructs in terms of high auto-correlations across time. Furthermore, these results may have been biased by not taking cross-sectional associations between symptom levels and QoL measured at the same time point into account.

### Aims of the study

To address the aforementioned limitations, in this study, we examined the prospective, reciprocal associations between negative symptoms, depression, anxiety, and QoL at three prospective assessment points (baseline, 6 months, 12 months) in a sample of patients with a severe psychotic disorder currently being treated with integrated care including a high fidelity variation of ACT, so-called Therapeutic Assertive Community Treatment (TACT). Analyses were carried out using cross-lagged panel models within the structural equation modeling framework [16] to test the hypothesis that QoL after 12 months is predicted by anxiety, depression, and negative symptoms while controlling for the stability of and cross-correlations between these constructs. Additionally, we hypothesized that the beneficial effect on QoL is mediated by negative symptoms, depressive symptoms, and anxiety.

## Materials and Methods

### Context

ACCESS is an integrated care program for people with non-affective and affective severe psychotic disorders that incorporates TACT within a multi-sectoral and interdisciplinary care network of inpatient and outpatient providers [8, 11, 17]. The effectiveness of the ACCESS program was assessed within these three studies so far: (a) the ACCESS I study assessed the implementation of the model [10, 14]; (b) the ACCESS II study assesses all patients entering the program since the approval by health insurances in Germany [11, 17, 18]; and (c) the ACCESS III study evaluated the effectiveness of the expansion of the model to adolescent (from the age of 12 years) and young adult patients in the early stage of the illness [8].

### Study design and sample

The ACCESS II study is a prospective, single-center, ongoing, long-term study assessing the effectiveness and efficiency of the so-called “Hamburg Model of Integrated Care (ACCESS)” for people with severe psychotic disorders [8, 11, 14, 17–20]. It investigates the long-term effectiveness of the identically named integrated care model ACCESS in a patient group diagnosed with affective or non-affective psychotic disorders also meeting the severe and persistent mental illness criteria. The ACCESS program is ongoing, 433 patients entered the program in the here studied enrollment period from May 2007 to September 2019. Those who participated in the program for at least 1 year (*n* = 418; 96.5% of the total enrollment) were included in the analysis. The trial was approved by the local ethics committee (number: PV4059) and is registered at ClinicalTrials.gov (identifier: NCT01888627).

### Inclusion and exclusion criteria

Inclusion criteria for the study are (a) aged 12 years or older, (b) presence of one of the following diagnoses according to the Diagnostic and Statistical Manual of Mental Disorders (DSM-IV-TR; [21]): schizophrenia, schizophreniform disorder, schizoaffective disorder, delusional disorder, psychotic disorder not otherwise specified, bipolar disorder most recent severe with psychotic symptoms, and major depression, single or recurrent, severe with psychotic symptoms; and (c) written informed consent by the patient (≥18 years) or by guardians with written informed assent by patient (12–17 years). Exclusion criteria comprised (a) presence of one of the following diagnoses according to DSM-IV-TR: alcohol- or substance-induced psychosis (comorbid alcohol, substance abuse, or dependence were tolerated), psychotic disorder due to a medical condition, and mental disability.

### Assessments and measures

Assessments were carried out at baseline, week 6, and months 3, 6, and thereafter every 6 months (13 examination times) by trained raters. All diagnoses were assessed as follows: (a) psychosis and comorbid mental disorders with the German version of Structured Interview I and, if indicated II for DSM-IV [22]; chronic somatic disorders, social support diagnoses (Z-diagnosis), and suicide attempt diagnoses with the ICD-10-GM [23]. Demographic characteristics were assessed with the Early Psychosis File Questionnaire [24] and psychopathology with the Brief Psychotic Rating Scale (BPRS; [25]). Here, item 2 of the BPRS was used to measure severity of anxiety and item 3 for severity of depression. Item 13 (self-neglect), item 16 (blunted affect), item 17 (emotional withdrawal), and item 18 (motor retardation) were used to form a summary score of these four negative symptoms according to Ref. [26]. Furthermore, functional level was assessed with the Global Assessment of Functioning Scale (GAF; [21]), severity of illness for schizophrenia spectrum disorders with the Clinical Global Impressions Scale-Schizophrenia (CGI-Sch; [27]), severity of illness for bipolar disorder (affective psychosis) with the CGI–Bipolar Disorder (CGI-BP; [28]), QoL with the Quality of Life Enjoyment and Satisfaction Questionnaire (Q-LES-Q-18; [29]). Q-LES-Q-18 [29] is a self-report instrument developed for patients with schizophrenia to assess their satisfaction with several life domains. Each of its 18 items is rated on a five-point Likert scale ranging from “not at all or never” to “frequently or all the time” depending on how often a person reports aspects of the QoL questions. Higher values indicate better QoL. In order to evaluate the questionnaire, the mean value is formed over all 18 items. The subscales (physical health, subjective feelings, leisure time active ties, and social relationships) are also evaluated by forming means (and standard deviations, SDs). In order to make the results easier to interpret and comparable, the mean values were transformed to a value range from 0 to 100 with higher values being associated with a higher self-reported QoL.

### Statistical analyses

Analyses were performed with SPSS version 25 and Mplus version 8.0 [30]. Descriptive analyses consisted of frequencies in categorical variables and means and SDs for continuous variables. Bivariate correlations among model variables were calculated across the three time-points (baseline [T0], 6 months [T1], and 12 months [T2]).

Effect sizes were expressed as correlation coefficients and Cohen’s *d* for pre–post, pre–follow-up, and post–follow-up assessments ([*M*_post_ – *M*_pre_]/*SD*_pre_) for the descriptive analyses, and standardized partial regression coefficients for the cross-lagged panel models. Cohen’s cut offs for small, medium, and large effects were set at ≥0.2, 0.5, and 0.8, respectively. Similarly, for correlation analyses correlation coefficients of ≥0.1, 0.3, and 0.5 were used to indicate weak, moderate, and strong correlations, respectively.

Cross-lagged panel models based on the three assessment points (baseline [T0], 6 months [T1], and 12 months [T2]) were calculated to investigate the longitudinal relationships between negative symptoms, depressive symptoms, anxiety, and QoL. These models allow estimating the reciprocal relationships between model variables by using earlier measures of a construct to predict later measures of another construct, that is, cross-lagged association. Simultaneously, the stability of each construct is estimated by regressing earlier measures of a construct on later measures of a construct, that is, autoregressive effect [31]. Residual variances were allowed to correlate at same assessment points. The significance of the indirect effect was tested by calculating bootstrapped, bias-corrected confidence-intervals with 1,000 iterations of the indirect effect. Missing values were handled with use of Full Information Maximum Likelihood. Model fit was assessed by the Comparative Fit Index (CFI), the Tucker–Lewis index (TLI), and the Root-Mean-Square Error of Approximation (RMSEA). A good-fitting model should produce CFI- and TLI-values higher than 0.95, and a RMSEA-value lower than 0.05.

## Results

### Sociodemographic and illness characteristics at baseline

Sociodemographic and illness characteristics at baseline of the 418 patients are displayed in Table 1. Both genders were almost equally represented in the patient cohort (47.8% male and 52.2% female). Over two-thirds (70.1%) were diagnosed with non-affective psychosis. Schizophrenia was the most frequent diagnosis (60.3%), followed by bipolar I disorder (14.8%) and schizoaffective disorder (13.6%). About 27.5% were included during their first episode, whereas 72.5% had already experienced at least one or multiple prior episodes. Concurrent with meeting the SMI criteria, patients displayed high scores of psychopathology (BPRS mean = 78.47%), severity of illness (CGI-S total mean = 5.53, SD = 0.93), and low functioning level (GAF mean = 39.51, SD = 12.48), as well as low QoL-related scores (Q-LES-Q-18 total mean = 36.98, SD = 17.99) at baseline.

**Table 1.** Demographic and psychopathological characteristics of the sample at baseline (T0).

	*N* (%*)*	Mean	SD
Patient characteristics			
Age	418	36.17	14.03
Gender			
Male	200 (47.8%)	–	–
Female	218 (52.2%)	–	–
Diagnosis and phase of illness			
Diagnosis			
Affective psychosis	125 (29.9%)	–	–
Non-affective psychosis	293 (70.1%)	–	–
Diagnostic distribution			
Schizophrenia	252 (60.3%)	–	–
Bipolar I disorder	62 (14.8%)	–	–
Schizoaffective disorder	57 (13.6%)	–	–
Others	47 (11.3%)	–	–
Phase of illness			
First episode	115 (27.5%)	–	–
Multiple episode	303 (72.5%)	–	–
Severity of illness			
CGI-S total	398 (95%)	5.53	0.93
CGI-S depression	398 (95%)	4.24	1.28
CGI-S cognitive	398 (95%)	4.22	1.31
CGI-S positive	398 (95%)	4.82	1.64
CGI-S negative	398 (95%)	4.11	1.44
Psychopathology			
BPRS	418 (100%)	78.47	20.78
Functioning level			
GAF	397 (95%)	39.51	12.48
Quality of life, Q-LES-Q-18			
QoL total score	382 (91%)	36.98	17.99
Subscore physical health	383 (92%)	34.92	19.23
Subscore subjective feelings	382 (91%)	39.66	21.87
Subscore leisure time activities	381 (91%)	34.26	23.44
Subscore social relations	382 (91%)	36.33	19.65

*Abbreviations:* BPRS, Brief Psychiatric Rating Scale; CGI, Clinical Global Impression Scale; GAF, Global Assessment of Functioning Scale; Q-LES-Q18, Quality of Life Enjoyment and Satisfaction Questionnaire (scores are transformed from 0 to 100); QoL, quality of life; *SD*, standard deviation.

Table 2 shows the BPRS and Q-LES-Q-18 baseline and changes scores over 1-year in level of total psychopathology, negative symptoms, depression, anxiety, and QoL. Over the first 6 months, there was a highly significant improvement in overall psychopathology, negative, depressive, anxiety symptoms, and QoL. The effect was small to medium for negative and depressive symptoms (*d* = 0.39–0.63), and large for overall psychopathology, anxiety and QoL (total score, anxiety and QoL: *d* = 1.11–1.36). Between 6-month and 12-month follow-up level of symptomatology and QoL did not change significantly.

**Table 2.** Means, standard deviations, and changes over time in BPRS and Q-LES-Q-18.

	T0	T1	T2	T0–T1	*t*	*p*	Cohens *d*	T1–T2	*t*	*p*	Cohens *d*
	*M* (*SD*)	*M* (*SD*)	*M* (*SD*)	*M* (*SD*)	(T0–T1)	(T0–T1)	(T0–T1)	*M* (*SD*)	(T1–T2)	(T1–T2)	(T1–T2)
BPRS total score	78.47	50.18	49.38	28.30	25.357	<0.001	1.36	0.64	1.064	0.288	0.061
	(20.78)	(12.9)	(13.54)	(20.85)				(10.48)			
BPRS 2 anxiety	4.67	2.95	2.96	1.78	21.134	<0.001	1.11	0.01	0.14	0.889	0.008
	(1.5)	(1.19)	(1.16)	(1.60)				(1.19)			
BPRS 3 depression	4.02	2.97	2.97	1.05	12.009	<0.001	0.63	−0.01	−0.184	0.854	−0.01
	(1.63)	(1.05)	(1.17)	(1.66)				(1.21)			
BPRS 13 self-neglect	3.22	2.23	2.22	1.02	11.441	<0.001	0.60	0	0.062	0.950	0.003
	(1.82)	(1.23)	(1.3)	(1.69)				(0.9)			
BPRS 16 blunted effect	3.65	2.95	2.92	0.68	7.478	<0.001	0.39	0.06	1.023	0.307	0.057
	(1.7)	(1.17)	(1.25)	(1.73)				(1.09)			
BPRS 17 emotional withdrawal	4.16	3.14	3.20	0.99	9.987	<0.001	0.53	−0.04	−0.63	0.529	0.035
	(1.75)	(1.43)	(1.36)	(1.88)				(1.15)			
BPRS 18 motor retardation	2.83	1.92	1.85	0.91	9.947	<0.001	0.53	0.08	1.429	0.154	0.08
	(1.72)	(1.12)	(1.12)	(1.72)				(0.98)			
Q-LES-Q-18 total score	2.48	3.23	3.30	−0.75	−20.121	<0.001	−1.11	0.05	1.321	0.188	0.076
	(0.72)	(0.61)	(0.68)	(0.80)				(0.59)			

*Abbreviations:* BPRS, Brief Psychiatric Rating Scale; *M*, mean; Q-LES-Q18, Quality of Life Enjoyment and Satisfaction Questionnaire; *SD*, standard deviation; T0, baseline; T1, 6 months; T2, 12 months.

### Correlations between model variables

As shown in Table 3, model variables were significantly correlated with effect sizes ranging between weak (0.11) and strong (0.69). Exceptions mainly involved level of anxiety. In detail, no significant associations were found for anxiety at baseline with negative symptoms (6 months and 12 months), depression and QoL (12 months) as well as between anxiety at 6-month follow-up and severity of negative symptoms at baseline. Furthermore, QoL at baseline was not significantly associated with severity of depression at 6-month follow-up.

**Table 3.** Bivariate correlations between model variables.

Model variables	Assessment timepoints										
	1	2	3	4	5	6	7	8	9	10	11
Negative symptoms T0	–										
Negative symptoms T1	0.36***	–									
Negative symptoms T2	0.35***	0.69***	–								
Depression T0	0.42***	0.16**	0.18**	–							
Depression T1	0.15**	0.48***	0.28***	0.28***	–						
Depression T2	0.18**	0.34***	0.52***	0.25***	0.40***	–					
Anxiety T0	0.11*	0.03	−0.06	0.26***	0.14**	−0.01	–				
Anxiety T1	0.11	0.33***	0.21***	0.16**	0.44***	0.25***	0.30***	–			
Anxiety T2	0.12*	0.28***	0.40***	0.16**	0.26***	0.57**	0.15**	0.48***	–		
QoL T0	−0.17**	−0.27***	−0.24***	−0.17**	−0.10	−0.15*	−0.14**	−0.04	−0.10	–	
QoL T1	−0.16**	−0.38***	−0.32***	−0.17**	−0.46***	−0.31***	−0.11*	−0.38***	−0.29***	0.25***	–
QoL T2	−0.16**	−0.37***	−0.51***	−0.14*	−0.26***	−0.59***	0.04	−0.24***	−0.52***	0.26***	0.59***

*Note.* Table shows correlation coefficients assessed at baseline (T0), after 6 months (T1) and after 12 months (T2).

*Abbreviation:* QoL, quality of life.

*p < 0.05, **p < 0.01, ***p < 0.001.

### Results of the cross-lagged panel model

**Figure 1.**
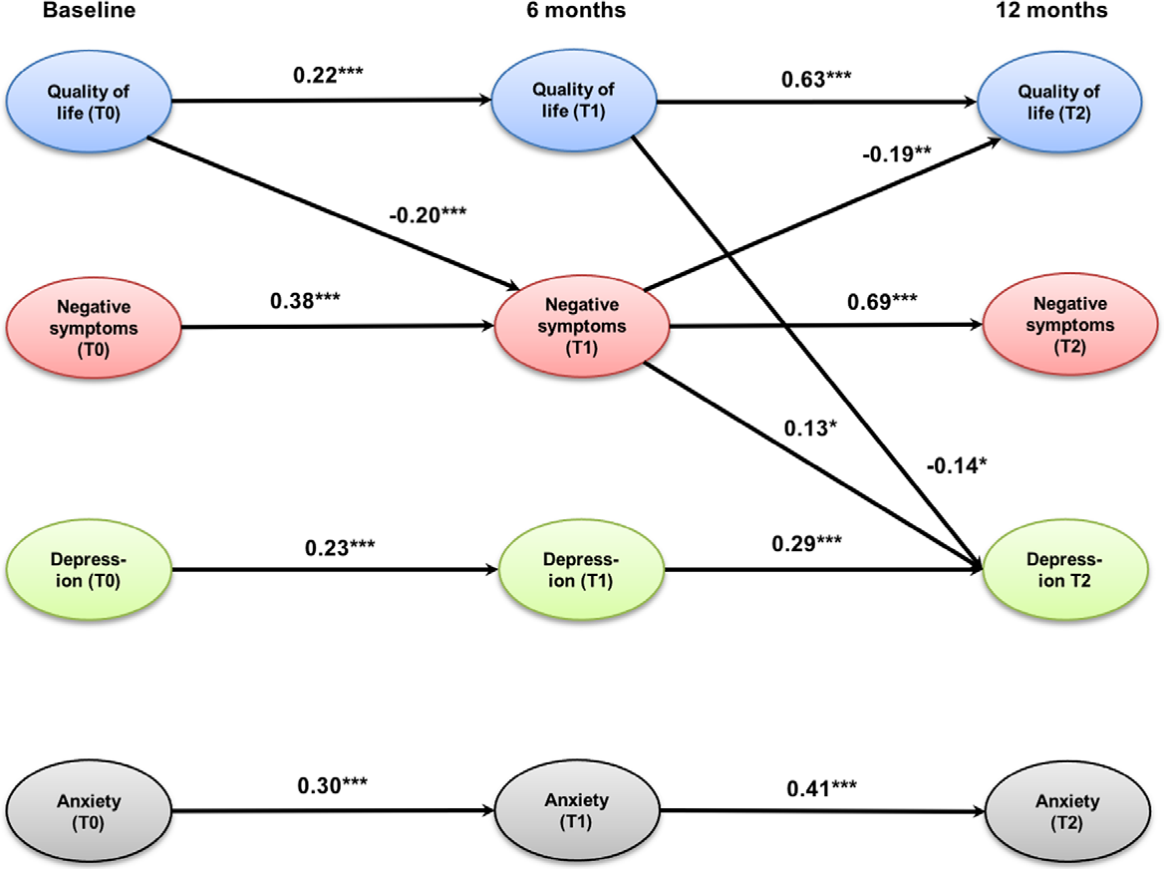
Cross-lagged panel model of the relationships between quality of life, negative symptoms (self-neglect, blunted affect, emotional withdrawal, and motor retardation), depression, and anxiety. Only significant coefficients are displayed; values are standardized path coefficients. ***p < 0.001, **p < 0.01, *p < 0.05.

The cross-lagged panel model (Figure 1) showed an excellent fit to the data as indicated by the following fit indices: CFI = 0.99, TLI = 0.97, and RMSEA = 0.04 (0.00; 0.07; *p* = 0.59). QoL, negative symptoms, depression, and anxiety were all stable across time as indicated by significant auto-regression coefficients between 0.22 for QoL (T0–T1) and 0.69 for negative symptoms (T1–T2). All associations were stronger between 6 and 12 months (T1–T2) than between baseline and 6-month follow-up (T0–T1). QoL at baseline significantly predicted negative symptoms at 6-month follow-up, which predicted improvements in QoL at 12-month follow-up. This indirect, mediated effect was significant (95% CIs of standardized IE = 0.01; 0.08, *p* = 0.03). Improvements in both QoL and negative symptoms after 6 months significantly predicted improvements in depression at 12-month follow-up. The indirect effect from QoL at baseline to depression at 12-month follow-up through improvements of negative symptoms was small and reached only a trend-level (95% CIs of standardized IE = −0.07; −0.01, *p* = 0.08). The same applied to the indirect effect of QoL at baseline and after 6 months on depression after 12 months (95% CIs of standardized IE = −0.07; −0.01, *p* = 0.07). Anxiety could only be predicted by previous levels of anxiety, but had no significant association with any other model variable.

## Discussion

The ongoing ACCESS II trial assesses the effectiveness of the integrated care model, including TACT for people with severe psychotic disorders fulfilling established SMI criteria [8, 11, 17, 19]. The present study aimed to shed further light on the temporal relationships between QoL and levels of anxiety, depression, and negative symptoms as these have been demonstrated to be amenable to change through the IC treatment [8, 13, 14].

### Key findings

The cross-lagged panel model showed that prior levels of symptom severity and impairment in QoL predict subsequent levels at the following assessment point. Notably, stability among constructs was highest for negative symptoms and QoL between 6- and 12-month follow-up. Despite the relative stability of each construct over time, significant changes in variable levels could be shown between baseline and 6-month follow-up, whereas there was no significant change between 6- and 12-month follow-up. This could be due to a certain generalization or ceiling effect of the intervention.

Three main indirect pathways leading to improvements after 12 months were detected. First, higher levels of QoL at baseline led to fewer negative symptoms after 6 months which even yielded further improvements in QoL after 12 months. Second, this points to a mediating effect of negative symptoms on QoL over the course of a year. This mediating effect of negative symptoms on future QoL has not previously been recognized. This finding supports recent results based on the usage of change-scores that improvements in negative symptoms may be a relevant mechanism of change of ACT treatment [12]. This implies that in order to optimize effects of ACT on QoL, severity of negative symptoms should be reduced during the early phases of the intervention. Such interventions then allow improving QoL more than what would be expected, if the effect would be limited to the reduction of clinical symptoms.

High levels of negative symptoms tend to impair the social relations and general ability to participate in everyday life [32, 33] which in turn causes a lower level of perceived QoL [34]. This could explain the central role of negative symptoms as a mediating factor as shown by our model analysis.

In a third pathway, the reduction of depression after 12 months was achieved by improvements in negative symptoms after 6 months which in turn was determined by the level of QoL at baseline. However, this indirect effect is small and only reached a trend level. Notably, depression at 12-month follow-up was also predicted by a small indirect effect through improvements of QoL from baseline to 6-month follow-up. Interestingly, anxiety showed no association with other variables, although it significantly improved over time. This is in contradiction to previous results (e.g., [12]) but is in line with current guidelines suggesting that targeting depression and negative symptoms together may produce beneficial effects [35].

### Limitations

While our study has several strengths (e.g. large sample size, patient sample that is hard to be treated, long follow-up), several limitations need to be mentioned. All variables were measured by only one indicator. This made it necessary to use manifest instead of latent variables, which may have underestimated the path coefficients and the amount of explained variance in each dependent variable. Relatedly, we assessed depression and anxiety by only one single item and together with negative symptoms from the same instrument, which may have overestimated the correlations between them. In future studies, it is therefore recommended to assess these constructs by several assessments and different informants, for example, clinician-ratings and self-reports. Another limitation refers to the fact that we could use only three assessment points covering 1 year. It might be interesting in future studies to use more assessment points and over a longer time-period to better capture the dynamic nature between severity of symptoms and QoL. Moreover, other factors that have not been included into the model (e.g., level of functioning and social support) to diminish model complexity may also have an important impact on the assessed model variables [12].

### Clinical implications

Quality of life, negative, and depressive symptoms showed a reciprocal interaction during the course of treatment. Anxiety symptoms, on the other hand, seem to be less influenced by this interaction. It could be interpreted that anxiety symptoms are a part of the psychosis itself and are present continuously, seemingly without affecting QoL in a significant way so that treatments specifically targeting anxiety are necessary.

Each construct is quite stable over time, in particular between 6 and 12 months, with small to medium effect sizes. One possible explanation for the strong association between 6 and 12 months may be might be that it is due to a generalization effect of the intervention where the improvement from the intervention reaches a plateau at which the effect stabilizes [36]. Furthermore, QoL and negative symptoms tend to have a more stable course than anxiety and depression, which fluctuate on a daily or weekly basis [37, 38]. This is well in line with the result that improvements mainly took place between T0 and T1.

We detected three main pathways to improvements after 12 months that are only partially in line with previous literature: Level of QoL baseline leads to improvements in negative symptoms after 6 months which predicts larger improvements of quality of life after 12 months (=mediation effect). This implies clinically that one of the most efficient ways to improve QoL might be to target it directly but also to target negative symptoms as it has been done in ACT. Therefore, negative symptoms may be an important mechanism of change of ACT. This is well in line with our previous studies [11, 12, 19].

Improvements in QoL after 6 months predict improvements in depression after 12 months via improvements in negative symptoms. However, the indirect effect is small and only significant on a trend-level. It means that QoL at baseline determines the severity of negative symptoms after 6 months. Such improvements in negative symptoms may lead to improvements in depression as patients do not need to adopt negative symptoms as a dysfunctional coping strategy any longer to protect themselves from negative feedback from the social environment [12, 14, 19]. This sequence of variables (negative symptoms and depression) is in line with our previous work, but notably, depression had no effect on QoL [39–41].

## Summary

Since QoL, negative symptoms, and depressive symptoms influence each other, they should each be the target of therapeutic interventions. With regard to QoL, these are, in addition to the improvement of psychopathology, above all the social, personal, family, and occupational functioning level. Psychopathology has a major impact on the level of social functioning and is also associated with depression. Depression, in turn, negatively influences QoL.

Among the factors contributing to the reduction in negative and depressive symptoms during ACCESS-treatment the fact, that continuous psycho- and pharmacotherapy are ensured from early on plays an important role, as well as the active follow-up that the multi-professional team provides in situations of non-compliance, adverse life conditions, or missed appointments. The intense and comprehensive care that is provided for different medical and social needs, with the 24/7 possibility of contact while the patients’ everyday life can go on as an out-patient creates an environment, where negative and depressive symptoms are reduced, which in turn contributes to the improvement of QoL.

Taken together, our results suggest that, in particular, negative symptoms may function as a potential mechanism of change of integrated care in patients with SMI. Negative symptoms might be a major driver of non-adherence to therapy, which is one of the most important factors in continued psychopathology and its sequelae, such as decreased social and overall functioning, decreased QoL and increased depression. Therefore, in addition to targeting QoL, negative symptoms, anxiety, and depression directly, it seems especially promising to integrate interventions for QoL and negative symptoms to achieve better generalization effects on QoL and depression. Our results further propose that the ACT therapists could begin with the treatment of negative symptoms and QoL, which may then trigger or at least facilitate improvements in depressive symptoms and QoL after 12 months.

## Data Availability

The datasets used and/or analyzed during the current study are available from the corresponding author upon reasonable request.
